# Cis-regulatory mutations associate with transcriptional and post-transcriptional deregulation of gene regulatory programs in cancers

**DOI:** 10.1093/nar/gkac1143

**Published:** 2022-12-08

**Authors:** Jaime A Castro-Mondragon, Miriam Ragle Aure, Ole Christian Lingjærde, Anita Langerød, John W M Martens, Anne-Lise Børresen-Dale, Vessela N Kristensen, Anthony Mathelier

**Affiliations:** Centre for Molecular Medicine Norway (NCMM), Nordic EMBL Partnership, University of Oslo, 0318 Oslo, Norway; Department of Cancer Genetics, Institute for Cancer Research, Oslo University Hospital Radiumhospitalet, 0310 Oslo, Norway; Department of Medical Genetics, Institute of Clinical Medicine, University of Oslo and Oslo University Hospital, Oslo, Norway; Department of Cancer Genetics, Institute for Cancer Research, Oslo University Hospital Radiumhospitalet, 0310 Oslo, Norway; Centre for Bioinformatics, Department of Informatics, University of Oslo, Gaustadalléen 23 B, N-0373 Oslo, Norway; KG Jebsen Centre for B-cell malignancies, Institute for Clinical Medicine, University of Oslo, Ullernchausseen 70, N-0372 Oslo, Norway; Department of Cancer Genetics, Institute for Cancer Research, Oslo University Hospital Radiumhospitalet, 0310 Oslo, Norway; Erasmus MC Cancer Institute and Cancer Genomics Netherlands, University Medical Center Rotterdam, Department of Medical Oncology, 3015GD Rotterdam, The Netherlands; Department of Cancer Genetics, Institute for Cancer Research, Oslo University Hospital Radiumhospitalet, 0310 Oslo, Norway; Department of Cancer Genetics, Institute for Cancer Research, Oslo University Hospital Radiumhospitalet, 0310 Oslo, Norway; Department of Medical Genetics, Institute of Clinical Medicine, University of Oslo and Oslo University Hospital, Oslo, Norway; Centre for Molecular Medicine Norway (NCMM), Nordic EMBL Partnership, University of Oslo, 0318 Oslo, Norway; Department of Cancer Genetics, Institute for Cancer Research, Oslo University Hospital Radiumhospitalet, 0310 Oslo, Norway; Department of Medical Genetics, Institute of Clinical Medicine, University of Oslo and Oslo University Hospital, Oslo, Norway

## Abstract

Most cancer alterations occur in the noncoding portion of the human genome, where regulatory regions control gene expression. The discovery of noncoding mutations altering the cells’ regulatory programs has been limited to few examples with high recurrence or high functional impact. Here, we show that transcription factor binding sites (TFBSs) have similar mutation loads to those in protein-coding exons. By combining cancer somatic mutations in TFBSs and expression data for protein-coding and miRNA genes, we evaluate the combined effects of transcriptional and post-transcriptional alterations on the regulatory programs in cancers. The analysis of seven TCGA cohorts culminates with the identification of protein-coding and miRNA genes linked to mutations at TFBSs that are associated with a cascading trans-effect deregulation on the cells’ regulatory programs. Our analyses of cis-regulatory mutations associated with miRNAs recurrently predict 12 mature miRNAs (derived from 7 precursors) associated with the deregulation of their target gene networks. The predictions are enriched for cancer-associated protein-coding and miRNA genes and highlight cis-regulatory mutations associated with the dysregulation of key pathways associated with carcinogenesis. By combining transcriptional and post-transcriptional regulation of gene expression, our method predicts cis-regulatory mutations related to the dysregulation of key gene regulatory networks in cancer patients.

## INTRODUCTION

Dysregulation of the gene expression regulatory programs in a cell is a hallmark of cancer. The often observed aberrant gene expression in cancer can be triggered by deregulation at any regulatory level (transcriptional and post-transcriptional) (1,2). While the majority of studies have focused on the mutations lying within protein-coding regions, most alterations occur in the noncoding portion of the human genome, where cis-regulatory elements reside and act as genetic switches to ensure that gene expression occurs at correct times and intensities in the correct cells and tissues (3). Molecular alterations in these regions can modulate the entire regulatory network of the cells, conferring oncogenic traits associated with clinical and histopathological features in cancer (3). So far, identification of noncoding cancer driver events at cis-regulatory regions has been limited to few examples with high recurrence or high functional impact (3–7). Based on mutation recurrence along the human genome, the Pan-Cancer Analysis of Whole Genomes (PCAWG) consortium reported that patients harbor an average of ∼4.6 driver events in their tumors. The PCAWG consortium estimated that driver point mutations in noncoding regions (∼1.2 per patient) were less frequent than driver point mutations in protein-coding genes (∼2.6 per patient) (8). Large-scale discovery of noncoding drivers has been hindered by their low level of recurrence, the varying target size of functional elements, technical shortcomings, and their composite effect with small individual effect size on multiple regulatory regions, e.g. slightly altering, but not obliterating, protein-DNA interactions (4,8). Furthermore, while high-impact driver mutations are typically found and reported, medium-impact putative passenger mutations can have an aggregated effect on tumorigenesis, beyond the already annotated driver events (9).

Gene expression is mainly regulated at the transcriptional level by the binding of transcription factors (TFs) to promoters (cis-regulatory regions surrounding genes’ transcription start sites, TSSs) and enhancers (cis-regulatory regions distal to genes) at TF binding sites (TFBSs) (10,11). Most of the studies that predict noncoding driver mutations in cis-regulatory regions rely on the identification of mutational hotspots, which are regions with higher mutation frequencies than expected by chance (8,12–18). Other studies explore somatic mutations with a potential effect on TF-DNA interactions (19–22), based on DNA sequence information alone, and confirm the potential impact of the predicted mutations on gene expression by *in vitro* experiments. It has also been attempted to directly combine somatic mutation data with gene expression information to evaluate the impact of the mutations in cancer samples. For instance, causal cis-regulatory variations in breast cancers have been identified by differential allele-specific expression of genes between cancer and normal cells (23,24). Mutations close to the TSSs of genes were shown to exert an in-cis effect on the expression of the corresponding genes (25). A tool that can be used to associate mutations with changes in expression in gene networks is *xseq* (26). The tool was developed to predict mutations in protein-coding exons with trans-effect (26) and it was adapted to consider noncoding mutations associated with protein-coding genes in B cell lymphomas (27). This methodology specifically assesses the trans-associations between mutations and gene network expression alteration in cancer samples through either exonic or cis-regulatory mutations linked to protein-coding genes (26,27).

At the post-transcriptional level, one way to further control gene expression is through miRNAs acting as ‘buffers’ to induce translational repression and mRNA degradation (28,29). miRNA biogenesis generally occurs in mammals in three steps: transcription of a primary transcript (pri-miRNA) that can be several kilobases long, cleavage of the pri-miRNA into a precursor (pre-miRNA) of ∼70bp, and cleavage of the precursor to produce mature miRNAs of ∼22 bp (29,30). The mature miRNA sequence is then loaded in the RNA-induced silencing complex to specifically target mRNAs for repression through base-pair complementarity at the 3’UTR of mRNA targets. A miRNA sequence is predicted to target tens to thousands of mRNAs (31). The miRNA-mediated regulation of mRNA translation is not an on/off system but rather an interplay between miRNA-binding site specificity, and miRNA and mRNA abundance (28,32). Therefore, even small changes in miRNA abundance may affect the expression of several direct targets but also other mRNAs through a cascading effect, potentially leading to dysregulation patterns observed in cancer. This observation, amongst others, suggests that miRNAs can act as cancer drivers (33,34).

Despite active research on post-transcriptional regulation and the identification of miRNAs and their targets (35), the understanding of miRNA transcriptional regulation is currently limited (30). One obstacle was the lack of precise identification of pri-miRNA TSSs. The FANTOM5 consortium recently took advantage of the cap analysis of gene expression (CAGE) technology to identify pri-miRNA TSSs genome-wide from different cell types and tissues in human and mouse (36). Given their short size and the fact that they are not recurrently mutated (8), we hypothesize that the driver potential of miRNAs in cancer could be triggered by cis-regulatory mutations that alter their expression with a downstream cascading effect on the gene regulatory programs of the cancer cells.

The increasing data accumulation of high-quality direct TF-DNA interactions (37,38), pri-miRNA TSS locations (36), somatic cancer mutations and cancer cell expression data (39) provides an unprecedented opportunity to analyze alterations of gene regulatory programs in cancer by combining transcriptional and post-transcriptional levels of gene expression regulation. The PCAWG consortium stated that the community is facing a ‘paucity’ in the discovery of noncoding cancer drivers that could be improved by analyzing larger sample datasets (8). We hypothesize that focusing on regulatory variants within TFBSs associated with protein-coding and miRNA genes combined with gene expression data has the potential to pinpoint cis-regulatory variants linked to the dysregulation of key gene regulatory networks in cancer patients.

To this end, we adapted the framework of the *xseq* tool to predict cis-regulatory somatic mutations associated with the dysregulation of gene networks by considering both protein-coding and miRNA genes. We predict genes associated with cis-regulatory mutations with cascading trans-effects on the gene regulatory program alteration across seven cancer patient cohorts from The Cancer Genome Atlas (TCGA) (39). This analysis reveals 12 mature miRNAs recurrently associated with cis-regulatory somatic mutations in different cohorts. Functional enrichment analyses of the dysregulated networks downstream of the predicted protein-coding and miRNA genes confirm that pathways known to be associated with carcinogenesis are recurrently disrupted. We conclude that the interpretation of noncoding mutations can be improved by focusing on TF-DNA interactions with the combined analysis of both transcriptional and post-transcriptional regulation of gene expression to revert the paucity in the discovery of cancer-associated noncoding events.

## MATERIALS AND METHODS

All analyses were performed using the hg19 human genome assembly. When data were obtained from another human genome assembly, coordinates were converted to the hg19 assembly using the liftOver tool provided by the UCSC Genome Browser (40,41).

### Cancer patient data

We considered TCGA (39) cohort samples for which trios of (i) whole genome somatic mutations, (ii) RNA-seq, and (iii) small RNA-seq data were available with at least 30 patients per cohort. Data were downloaded from the International Cancer Genome Consortium (ICGC) portal (42) through the *icgc-get* client (Additional file 5). Altogether, we collected data for 349 samples from seven TCGA patient cohorts (35–89 donors per cohort; Additional file 1): BRCA-US (breast invasive carcinoma), HNSC-US (head and neck squamous cell carcinoma), LIHC-US (liver hepatocellular carcinoma), LUAD-US (lung adenocarcinoma), LUSC-US (lung squamous cell carcinoma), STAD-US (stomach adenocarcinoma), and UCEC-US (uterine corpus endometrial carcinoma).

We retrieved data from 256 samples collected by the ICGC Breast Cancer Working group (43,44) for which trios of whole genome somatic mutations, RNA-seq, and miRNA microarray data were available (Additional file 4). miRNA expression was measured using the Human miRNA Microarray Slide (Release 19.0) with Design ID 046064 (Agilent Technologies, Santa Clara, CA, USA; see (43) for details).

Somatic single nucleotide variants (SNVs) and small insertions and deletions (indels) called by MuSE (45) were retrieved from the ICGC portal for TCGA samples. For ICGC samples, we retrieved SNVs and indels called by the tools CaVEMan (46) and Pindel (47), respectively, used in the original study (43).

### RNA-seq and small RNA-seq normalization

Both RNA-seq and small RNA-seq raw counts were filtered to remove all genes with 0 reads in >50% of the samples for a given cohort. For each cohort, both matrices (RNA-seq and small RNA-seq) of raw counts were normalized to counts per million (cpm) using the *cpm* function from the R package edgeR (48) and the cpm values were scaled by log_2_ conversion. To avoid zeros, we added a pseudo-count of 1. Note that small RNA-seq reads were mapped to pre-miRNA coordinates by TCGA, providing information about pre-miRNA expression and not mature miRNAs.

The normalized microarray miRNA expression matrix for ICGC samples was retrieved from the original study where normalization was performed using the 90th percentile methodology (43). We used the normalized RNA-seq matrix provided by the ICGC Breast Cancer Working Group (43).

### Copy number alteration computation

We downloaded copy number alteration (CNA) values predicted using the GISTIC2 tool (49) for TCGA samples through the Firebrowse database at http://firebrowse.org (Additional file 5). ICGC CNA estimates were computed using ASCAT (v2.1.1) (50) and converted into GISTIC format with -2 for homozygous loss (nMinor + nMajor = 0), –1 for hemizygous loss (nMinor + nMajor = 1), 0 for normal (nMinor + nMajor = 2), 1 for three copies (nMinor + nMajor = 3), and 2 for more than three copies (nMinor + nMajor > 3). The CNA values assigned to the protein-coding genes were used in the *xseq* analysis to remove cis-effects of CNAs on the gene expression dysregulation assessment (26).

### Mutation rate analysis

For each sample, we calculated the mutation rates by dividing the number of mutated nucleotides within a set of regions (TFBSs, exons, and flanking regions) by the number of nucleotides covered by the given set of regions. TFBS genomic positions were obtained from UniBind (38) (see below). Protein-coding exon coordinates were retrieved from RefSeq Curated (51) (Additional file 5). Flanking regions were computed by (i) extending TFBS or exonic regions by 100, 500 and 1000 nucleotides on both sides using the *flank* bedtools subcommand and (ii) removing regions overlapping TFBSs and exonic regions using the *subtract* bedtools subcommand. Sets of regions were independently merged using the *merge* subcommand of the bedtools (52).

Random expectations for mutation rates were computed using 150 random sets of somatic mutations and applying the mutation rate computation described above. The random sets of mutations were generated by shuffling the original coordinates within the same chromosomes using the *shuffle* subcommand of the bedtools with the *-chrom* option.

miRNAs

Genomic coordinates of human pre-miRNAs were retrieved from miRBase v20 (53) and used to predict miRNA TSSs from CAGE data by the FANTOM5 consortium (36). When miRNA names in the miRNA-related files (expression, survival, cancer-associated miRNAs) used in this study were mapped to older versions of miRBase (starting from version v10), we updated the names according to the miRBase version (v22) using the miRBaseConverter R/Bioconductor package (54).

### Transcription factor binding sites

TFBSs were retrieved from the UniBind database (2019 version) at https://unibind.uio.no (38) (Additional file 5). The TFBSs correspond to high confidence direct TF-DNA interactions with both experimental (through ChIP-seq) and computational (through position weight matrices (PWMs) from JASPAR (55)) evidence (37,38). Indeed, these TFBSs were predicted with high PWM scores and proximity to ChIP-seq peak summits and were derived from 1983 ChIP-seq experiments for 231 TFs across 315 cell types and tissues (38).

### TFBS-gene association

We used the cis-regulatory element-gene associations from the GeneHancer database (v4.9), derived from eight sources to associate TFBSs to genes (Additional file 5; Supplementary Figure S8) (56). TFBSs overlapping a cis-regulatory element annotated in GeneHancer were associated with the corresponding gene in GeneHancer. TFBSs not overlapping annotated elements were associated with the closest TSS (for a protein-coding or a miRNA gene). We considered TSSs associated with protein-coding genes from RefSeq Curated (51) and TSSs associated with miRNAs by FANTOM5 (36). With this approach, about half of the TFBSs were associated with protein-coding or miRNA genes using GeneHancer associations and the other half with the closest TSS.

### TFBS mutations

Somatic mutations were intersected with TFBS locations using the *intersect* subcommand of bedtools v2.25.0 (52). All mutations in TFBSs associated with miRNAs were considered for the *xseq* analysis (see below). For mutations in TFBSs associated with protein-coding genes, we followed the approach previously used by Mathelier *et al.* for the *xseq* analysis (27). Specifically, we restricted the analysis to mutations associated with genes potentially dysregulated in the corresponding samples. Following (27), genes were considered as potentially dysregulated in a given sample in cohort *C* if its expression value *v* satisfied *v* < μ-1σ or *v* > μ+1σ (i.e. z-value< -1 or z-value > 1) where μ and σ correspond to the mean and standard deviation of the expression values of the gene in *C*.

### Loss-of-function mutations

Following Ding *et al.* (26) for protein-coding exonic regions, we considered only LoF mutations that are either (i) nonsense mutations (disruptive in-frame deletion, disruptive in-frame insertion, stop gained, start lost, stop lost, and stop retained variant), (ii) frameshift mutations (frameshift variant, initiator codon variant) or (iii) splice-site mutations (splice region variant, splice donor variant, splice acceptor variant). The analysis was performed using somatic mutation data obtained from whole exon sequencing in the same TCGA samples as for the other analyses.

### Protein-coding gene networks

Protein-coding gene networks were retrieved from (26) and were composed of 898,032 interactions. Briefly, the networks were constructed by combining gene associations from STRING v9.1 functional protein association (57), KEGG pathway datasets (58), WikiPathway (59) and BioCyc (60) as integrated into the IntPath database (61), and TF-target links from ENCODE (62) (see (26) for more details). We updated the weights of the connections whenever possible using the methods provided in *xseq*, following the methodology described in (26). Specifically, the original weight between a given gene *g* and a biological partner gene *p* was updated to 1 if *p* was found differentially expressed (Benjamini–Hochberg adjusted *P*-value ≤ 0.05) in samples where *g* is mutated in the same cohort (see Materials and methods in (26) for details). If there existed such genes *p*, then only these genes were kept connected to *g*. Original weights were kept otherwise.

### miRNA–target networks

miRNAs were associated with potential target protein-coding genes using predictions from TargetScan v7.2 (31). From the list of targets for each miRNA, we filtered out the targets with less than two predicted binding sites for the given miRNA to reduce false positives (63,64). miRNA-target weights were computed as *t_score* / 100 where *t_score* corresponds to the *targetScan context++* score percentiles from TargetScan. We updated the weights of the connections whenever possible following the same strategy as for protein-coding genes (see above).

### xseq analyses

The likely associations between mutations and dysregulation of protein-coding gene or miRNA target networks were calculated with *xseq* (26). This method requires the following as input: a gene expression matrix of samples (RNA-seq matrix), a binary sample-gene mutation matrix (a matrix indicating that a particular gene in a given sample is associated with a mutation), and a weighted network of connected genes. Taking advantage of the gene expression information, the method identifies genes in the sample-gene matrix whose biological partners (from the biological network) have expressions that deviate from neutral. This is computed by decomposing the expression distribution of each connected gene into three components (or regulatory status, namely, downregulation, neutral, and upregulation). Enrichment of both upregulated and downregulated genes within a set of biological partners is evaluated in individual samples and then across a cohort using a Bayesian hierarchical network. *xseq* outputs posterior probabilities associated with: (i) a sample-specific gene regulatory status (GRS, the probability of a given gene being dysregulated in a sample) for each gene connected to the gene associated with a mutation in a given sample, (ii) a sample-specific dysregulation probability (SSD, the probability that a mutation in a given gene in a given sample is associated with dysregulation of the gene's network) and (iii) a dysregulation across the cohort probability (DAC, the probability that mutations linked to a gene are associated with the dysregulation of its network across patients) (Supplementary Figure S9). In a first step, we removed lowly expressed genes in a cohort following the approach described by Ding *et al.* (26). Briefly, *xseq* considers the 90th percentile of expression for each gene and decomposes the distribution of these values into two Gaussian distributions corresponding to low and high expression values. We considered for further analysis the genes for which their 90th percentile of expression values lie within the high expression distribution with a posterior probability ≥0.8 (see Ding *et al.* (26) for details). Next, *xseq* was used to compute all the posterior probabilities to predict genes and cis-regulatory mutations in the cancer patient cohorts.

### Selection of predicted genes

We considered cohort-specific FDR computation to predict miRNAs and protein-coding genes. Specifically, we generated a set of 100 random controls for each cohort where the original network and the gene-sample association tables were shuffled; the RNA-seq matrix was not shuffled. For the biological networks, we kept the original number of edges, but both the target genes and their connection weights were shuffled. *xseq* was applied to each random control independently and the results of the 100 controls were aggregated to compute the threshold *t* on DAC that corresponds to an FDR of 0.05. If the corresponding threshold on the DAC cohort-specific posterior probability was <0.5, we chose 0.5 as the threshold. We considered genes with DAC above this threshold and SSD ≥0.5 in at least two samples as potential cancer-associated genes.

### Dysregulation heatmaps

The dysregulated networks for predicted protein-coding and miRNA genes are visualized as heatmaps where columns correspond to mutated samples and rows to connected genes. Heatmaps were constructed with connected genes dysregulated (GRS ≥ 0.5) in at least one sample with SSD ≥0.5. These genes are referred to as dysregulated genes.

### Aggregated and sample-specific networks

To evaluate whether the protein-coding genes predicted by cis-regulatory mutations are connected in the filtered networks (see Protein coding gene networks section), we built an aggregate network using all the predicted protein-coding genes within a cohort. We counted the number of clusters using the R packages *igraph* (65) and *ggnetwork* (66). Similarly, we built sample-specific networks and counted the number of clusters in each sample, only considering the predicted genes with DAC ≥*t* (with *t* being the threshold on the DAC that corresponds to an FDR of 0.05, see above) and SSD ≥0.5.

### Functional enrichment analysis

Given a list of dysregulated genes, functional enrichment analyses were performed using the R package enrichR (67) for the following databases: KEGG_2021_Human, WikiPathways_2019_Human, GO_Biological_Process_2021 and Panther_2016.

### Enrichment for cancer-associated genes and TFs

Given a set of genes, we assessed their enrichment for cancer-associated genes or TFs using hypergeometric tests using the *stats::phyper* function in R. The list of cancer protein-coding genes considered was constructed by considering genes that appear in at least two of the following databases: Network Cancer Gene (68), inToGen (69) and Cancer Gene Census (70). Cancer miRNA genes were retrieved from miRCancer (71) with data from 1 May 2019. TF genes were retrieved from the human transcription factor database (11).

### Survival analysis

To test whether miRNA expression was associated with survival, we used the METABRIC breast cancer cohort (72) with miRNA microarray expression (73) available for 1282 tumors. Expression values were downloaded from the European Genome-Phenome Archive, www.ebi.ac.uk/ega, accession number EGAS00000000122. Follow-up data were available from Curtis *et al.* (72). Kaplan–Meier survival analyses and log-rank tests were performed using the R package *survival* with tumors separated into ‘high’ or ‘low’ miRNA expression groups depending on expression values above or below the median. *P*-values were adjusted for multiple testing according to the Benjamini–Hochberg method.

### Results accessibility

The analysis with all the scripts and parameters can be found through the following link: https://bitbucket.org/CBGR/workspace/projects/DYS. We provide (i) the source code for the analysis at https://bitbucket.org/CBGR/dysmir_manuscript/src/master/ and (ii) a pipeline for users to run similar analyses with their data at https://bitbucket.org/CBGR/dysmir_pipeline/src/master/.

## RESULTS

### Transcription factor binding sites harbor a similar mutational load as protein-coding exons

We study the occurrence of somatic mutations from whole genome sequencing of 349 samples from seven cancer patient cohorts (35–89 samples per cohort) covering seven distinct cancer types from TCGA (39) (Additional files 1–2). Specifically, we select samples where trios of somatic mutations, RNA-seq, and small RNA-seq data are available. In aggregate, we examine 11 434 931 somatic single nucleotide variants and small insertions and deletions (from 2832 to 1 014 969 per sample; Additional file 2; Supplementary Figure S1).

**Figure 1.**
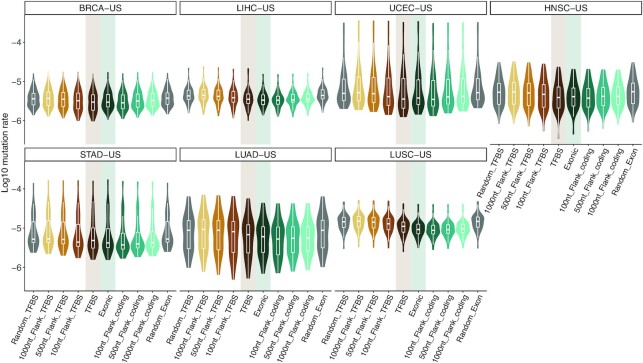
Comparison of mutation rates in TFBSs and exons versus their flanking regions and random mutation rates. Each panel corresponds to a specific cancer cohort (see title boxes) and each point corresponds to a sample. On each panel, the two central boxplots (shadowed) represent mutation rates in TFBS and exonic regions, the remaining box plots correspond to mutation rates in increasing-size flanking regions (100, 500 and 1000 nt) and mutation rates expected by chance (150 randomly distributed sets of mutations in the genome; Material and Methods).

To identify cancer-associated cis-regulatory mutations, we consider a set of TFBSs predicted as direct TF-DNA interactions in the human genome and stored in the UniBind database (38). These TFBS predictions are supported by both experimental (based on ChIP-seq) and computational evidence (based on position weight matrices) of direct TF–DNA interactions (see Materials and Methods and references (37,38) for details). We first assess whether this set of TFBSs represents regions of functional interest similar to the coding portion of the human genome commonly studied to predict cancer-associated mutations. These TFBSs cover ∼2.2% (68 071 257 nt) of the human genome, close to the exonic coverage of protein-coding genes (∼2.6%; 81 416 464 nt). Focusing on the somatic mutations, we observe that 1–2% of the mutations in each sample lie within these TFBSs (median of 277 mutations per sample; Additional file 2; Supplementary Figure S2). Mutation rates in TFBSs vary between cancer cohorts but are similar to the mutation rates observed in exons (two-tailed Wilcoxon tests *P*-values between 0.13 and 0.96; Figure 1 and Supplementary Figures S3 and S4). TFBSs are less mutated than their flanking regions (Figure 1). Note that regions of 1 kb surrounding TFBSs harbor mutation rates similar to what is expected by chance (two-tailed Wilcoxon tests *P*-values between 0.56 and 0.95; Supplementary Figures S3 and S4). While exons exhibit mutation rates similar to those observed within TFBSs (Supplementary Figure S5), their flanking regions show a smaller increase in mutation rates than the increase detected in the vicinity of TFBSs (Supplementary Figures S6 and S7).

Taken together, these results confirm that the noncoding mutation frequencies in the studied set of TFBSs follow a similar pattern to what is observed in protein-coding exons. It provides *a posteriori* confirmation that the set of TFBSs we consider is likely composed of functional regions in the human genome and can be used to highlight cis-regulatory mutations of functional interest in cancer genomes.

### Cis-regulatory and loss-of-function mutations complementarily alter protein-coding gene networks

We then seek to predict the cis-regulatory mutations that lie in these TFBSs and that lead to cascading effects on gene network deregulation, a hallmark of carcinogenesis. We first focus on the mutations in TFBSs linked to protein-coding genes and compare their effect on gene regulation to that of mutations altering the function of the protein-coding genes. We consider a protein-coding gene to be mutated through either a loss-of-function (LoF) somatic mutation in one of its exons as in (26) or a somatic mutation overlapping a TFBS associated with the gene. TFBSs are linked to protein-coding or miRNA genes based on cis-regulatory element-to-gene associations from GeneHancer (56) or distances to TSSs (Materials and Methods; Supplementary Figure S8). We estimate the potential trans-effect of the mutations on expression disruption in protein-coding gene networks using the *xseq* tool, following approaches implemented in previous studies (26,27). Specifically, the method uses a hierarchical bayesian approach to associate mutations with expression dysregulation in biological networks associated with the mutated protein-coding genes. In a nutshell, it assesses the posterior probability of the likely association between observing mutations in a set of patients and observed deviations from neutral expression in these samples for protein-coding genes in the same network. The likely trans-associations between mutations and gene network deregulation are first assessed in a sample-specific manner and then across samples from the same cohort (Supplementary Figure S9). Genes with low expression in a given cohort were filtered out; the distribution of the 90th percentile of expression for genes was decomposed into two Gaussian distributions corresponding to low and high expression values and only genes lying in the high expression distribution were retained (Materials and Methods). Furthermore, gene expression is corrected for copy number alterations (amplifications and deletions detected by GISTIC2 (49)) to compensate for copy number-related cis-effects on expression (Material and Methods). LoF mutations and mutations that overlap TFBSs are analyzed independently. Finally, we consider predictions that satisfy a false discovery rate (FDR) <0.05, computed empirically for each cohort using random controls (Materials and Methods).

**Figure 2.**
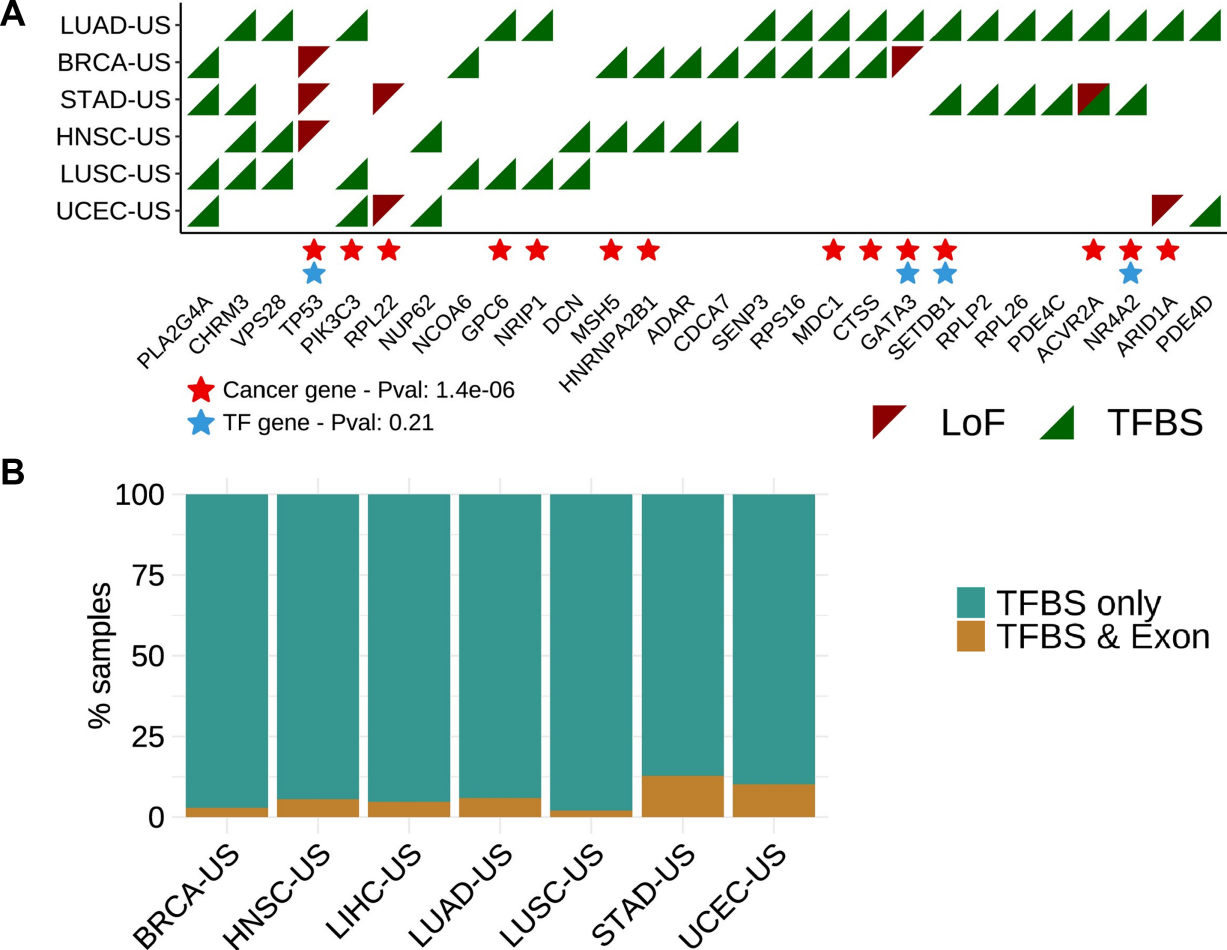
Pan-cancer predicted protein-coding genes. (**A**) Predictions are obtained applying the *xseq* tool when considering the effect on gene deregulation of protein-coding genes mutated through either LoF (red triangles) or cis-regulatory (TFBS; green triangles) mutations, independently. Genes, with mutations predicted to affect gene regulation in at least two cohorts are depicted here. Enrichment for cancer-associated genes (red stars) and TFs (blue stars) are evaluated using hypergeometric tests (p-values provided in the legend; Material and Methods). (**B**) Samples where genes are predicted with cis-regulatory mutations are considered for each cohort and assessed for the presence of LoF mutations in the same genes for the same cohort (TFBS & Exon) or no LoF mutation in the corresponding gene (TFBS only).

Out of the 7275 unique protein-coding genes linked to somatic mutations in the seven TCGA cohorts, 237 are associated with the deregulation of transcriptional networks in at least one cohort. Of these, 21 harbor LoF mutations (*TP53* and *RPL22* are predicted with LoF mutations in three and two cohorts, respectively; Figure 2A) and 219 are linked to cis-regulatory mutations associated to transcriptional deregulation (24 genes are found in more than one cohort; Figure 2A, Supplementary Figures S10 and S11, and Additional File 3). Three genes are linked to dysregulated networks in association with both LoF and cis-regulatory mutations but in different patients and/or cohorts: *ACVR2A*, *ARID1A* and *GATA3* (Figure 2A). These three genes are already known cancer drivers that we predict to be impacted by alternative mutational mechanisms (LoF or cis-regulatory mutations). The remaining genes are either associated with LoF or cis-regulatory mutations across cohorts (*TP53*, *RPL22* with LoF mutations; e.g. *PIK3C3* and *CHRM3* with cis-regulatory mutations; Figure 2).

From the combined list of 237 predicted protein-coding genes (Additional File 3), 81 are already annotated as cancer-associated genes (*P*-value = 9.3e–17; hypergeometric test) and 29 as TFs (*P*-value = 0.025; Supplementary Figures S10 and S11). We observe 28 genes to be predicted in at least two cohorts. These 28 genes are enriched for already known cancer-associated genes (*P*-value = 1.4e–6; hypergeometric test) but not for TFs (*P*-value = 0.21; hypergeometric test) (Figure 2A).

**Figure 3.**
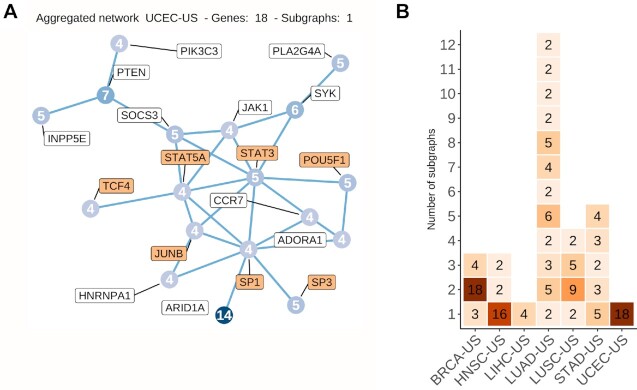
Predicted protein-coding genes linked to cis-regulatory mutations are connected in biological networks. (**A**) Subgraph detected among the predicted protein-coding genes in the Uterine Corpus Endometrial Carcinoma (UCEC-US) cohort. The number of samples in which each gene is predicted is shown within the nodes. TF names are highlighted with an orange background. (**B**) Heatmap showing the number of subgraphs (y-axis) found among the predicted protein-coding genes linked to cis-mutations in the TCGA cohorts (x-axis). The number of nodes within a subgraph is indicated in each cell. Genes not connected to any other predicted gene are not shown.

The genes predicted through cis-regulatory mutations rarely contained LoF mutation in the same tumors (Figure 2B and Supplementary Figure S12). We interpret this to mean that LoF and cis-regulatory mutations are possibly complementary mechanisms that alter the gene regulatory programs of cancer cells. We observe that multiple genes can be predicted through cis-regulatory mutations in the same sample. Furthermore, these genes tend to be interconnected in the dysregulated genes’ networks (Figure 3A). All these genes are predicted through mutations associated with cascading trans-effect in gene network dysregulation but the method cannot identify the specific main driver event or the combination of cis-regulatory mutations. When considering all the predicted genes per cohort, we detect a similar pattern with subnetworks of interconnected genes with a maximum of 12 subgraphs containing at least two nodes per cohort (mean = 3; median = 4.13; Figure 3B and Supplementary Figure S13). Altogether, these interconnected subnetworks suggest that the predicted genes are likely involved in similar biological pathways with altered expression associated with cis-regulatory somatic mutations.

### Deregulation of transcriptional activity and cancer pathways are trans-effect signatures of the predicted cis-regulatory and loss-of-function mutations

**Figure 4.**
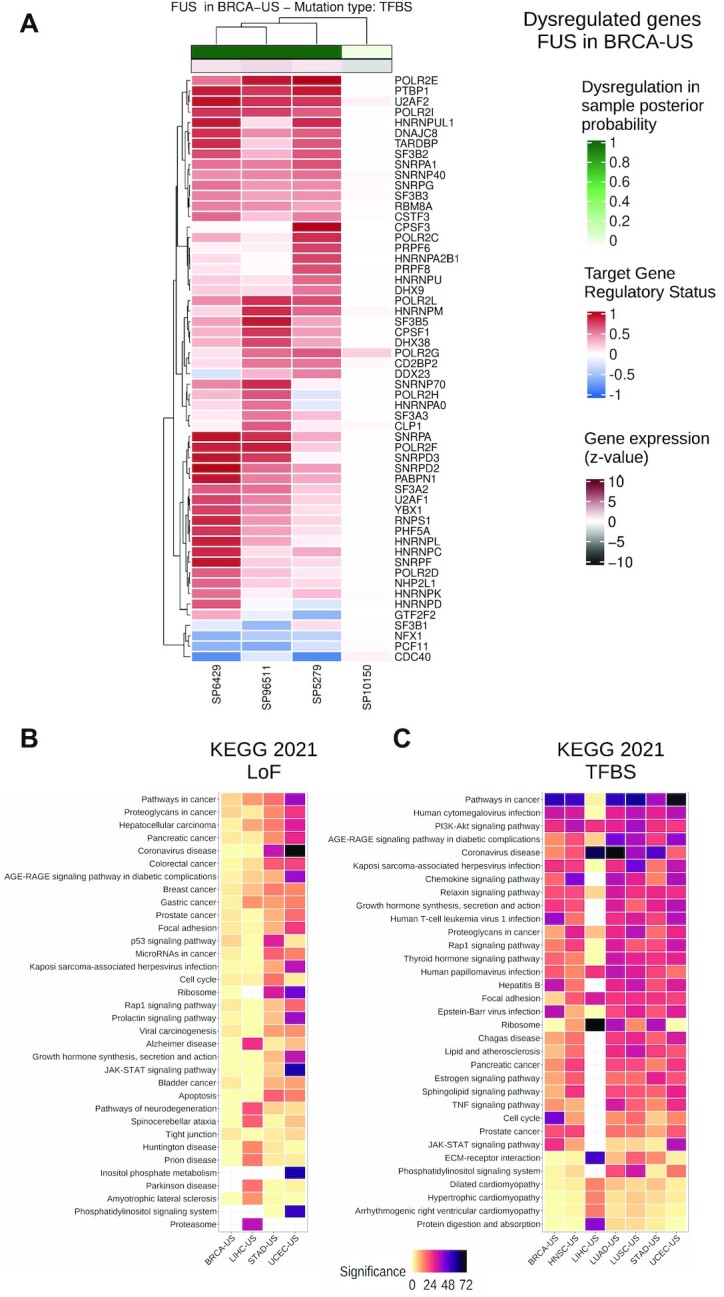
Dysregulated protein-coding gene networks and functional enrichment analysis. (**A**) Dysregulated gene network in samples where *FUS* is predicted through cis-regulatory mutations in breast cancer (BRCA-US) (rows: dysregulated genes associated with *FUS*; columns: samples with *FUS*-associated cis-regulatory mutations). The color scale represents the gene regulatory status posterior probability (red: up-regulation; blue: down-regulation—posterior probability * (–1)). The top horizontal bar shows the sample-specific dysregulation posterior probability computed by *xseq* for the samples harboring a cis-regulatory mutation in the *FUS* gene. The horizontal bar below shows the gene expression z-value of *FUS* (Materials and Methods). (**B**) KEGG 2021 most enriched terms computed from all the dysregulated genes associated with the predicted protein-coding genes (A is one example for *FUS*) by *xseq* with LoF mutations and (**C**) cis-regulatory mutations in TCGA cohorts (columns). Terms (rows) are ordered by their mean rank across all cohorts. Significance is provided as –log_10_(*P*-value).

To shed light on the functional role of the somatic mutations predicted to be associated with a cascading effect, we perform enrichment analyses on the altered gene expression profiles. One advantage of *xseq* is its capacity to highlight the specific genes in the biological networks that are dysregulated in the samples harboring the somatic mutations considered (Material and Methods) (26). These genes are consistently found to be either up- or down-regulated in the samples with predicted disrupted expression (see the blue and red colors in the upper and lower clusters in Figure 4A). These results highlight sets of genes up- or down-regulated across samples where cancer-associated genes are predicted.

We assess the biological relevance of the networks predicted to be dysregulated in association with either LoF or cis-regulatory mutations linked to the protein-coding genes. Functional enrichment analysis is performed using pathways from KEGG (58), WikiPathways (59) and Panther (74), and gene ontology biological processes (GO BP (75)) with the EnrichR tool (67). The dysregulated genes in the networks are enriched for transcriptional activity (‘regulation of transcription, DNA-templated’ from GO BP; Supplementary Figure S14). Combined with the enrichment of TFs in the complete list of predicted cancer-associated genes, this result emphasizes that the alteration of transcriptional regulation is likely a common feature of cancer cells throughout cancer types. Pathways already known to be associated with carcinogenesis (e.g. ‘Pathways in cancer’, ‘JAK-STAT signaling’, ‘PI3K-Akt signaling’, ‘p53 signaling pathway’, ‘Focal adhesion’ and ‘Apoptosis’; Figures 4B, C and Supplementary Figures S14–S17) are at the top of the enriched terms. The enrichment for cancer pathways confirms that our approach identifies somatic exonic and cis-regulatory mutations associated with potential protein-coding cancer-associated genes with cascading effect on regulatory alteration of key cancer-related pathways. Our results suggest that alteration of gene network expression could be achieved through cis-regulatory mutations associated with different genes in different patients but involved in the same pathways.

### Combining transcriptional and post-transcriptional regulation highlights pan-cancer miRNAs associated with gene expression alteration in tumors

The analysis of mutations linked to protein-coding genes presented above demonstrates that our methodology pinpoints cis-regulatory mutations likely associated with carcinogenesis. We hypothesize that our method could highlight cis-regulatory mutations linked to miRNAs with downstream cascading effects on the gene regulatory programs of the cells because miRNAs are involved in post-transcriptional regulation of gene expression. This novel approach of functional analysis of mutations aims to combine transcriptional (through mutations in TFBSs) and post-transcriptional (through regulatory networks of miRNA–targets) regulation to predict miRNAs associated with a trans-effect on gene expression alteration through somatic mutations in cis-regulatory elements.

Specifically, we adapt the *xseq* framework to infer cis-regulatory somatic mutations linked to miRNAs and associated with a cascading effect on miRNA target networks dysregulation. Similar to the analysis of protein-coding genes, we estimate the posterior probability of the likely association between the presence of mutations in TFBSs linked to a miRNA with observed deviations from neutral expression of the miRNA’s target genes. We consider miRNAs from miRBase (53) and their corresponding TSSs, which were identified using CAGE (Materials and Methods) (36). To assess the cascading effect of mutations linked to miRNAs on their targets’ expression, we examined the protein-coding genes predicted by TargetScan (31) to be targets of each miRNA. We limited the set of miRNA–target genes pairs to those where at least two target sites for the miRNA are predicted to reduce false positive predictions (63,64) (Materials and Methods). Note that we separately analyze miRNAs from both arms (5p and 3p) for each pre-miRNA sufficiently expressed in a TCGA cohort (Materials and Methods).

**Figure 5.**
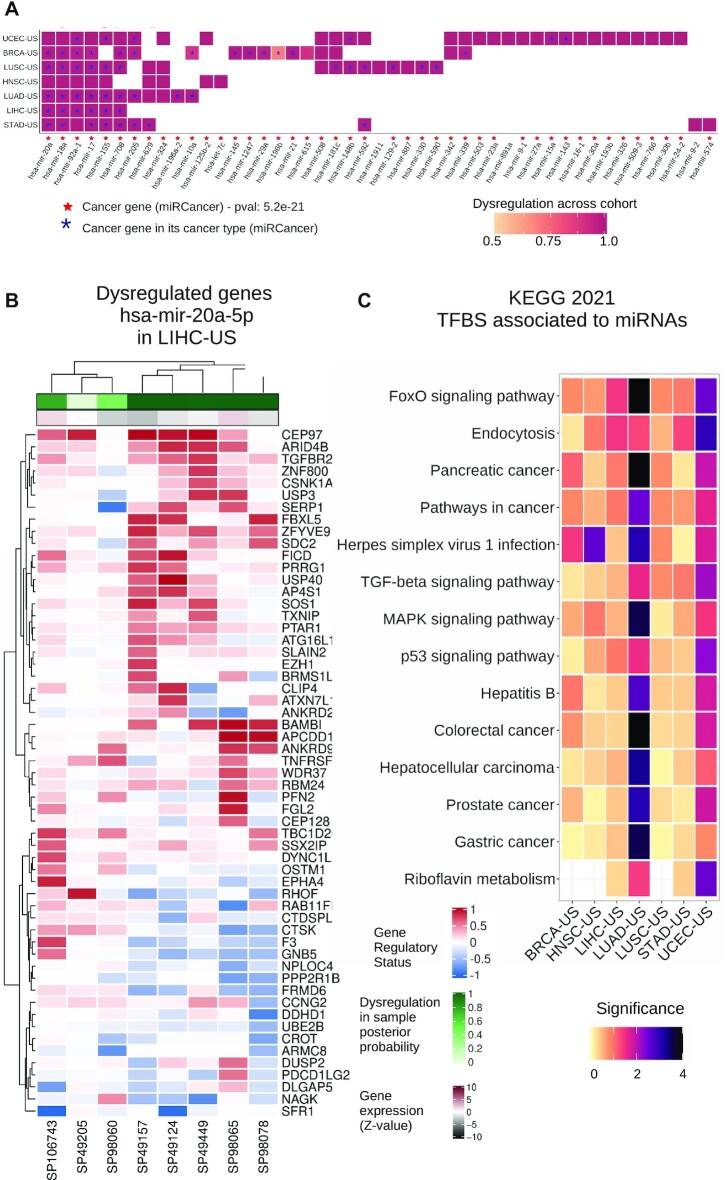
Overview of miRNA driver predictions and their dysregulated target networks. (**A**) Pre-miRNAs with mature miRNAs predicted as potential drivers by *xseq*. Cell colors indicate the posterior probability computed over the corresponding cohort. Red stars indicate that the miRNA is annotated as a cancer-associated miRNA in miRCancer (71). Blue stars indicate that the miRNA was reported as a cancer-associated miRNA in the specific cancer type where it is predicted by *xseq*, according to miRCancer annotation. (**B**) Dysregulated network of target genes for miRNA hsa-mir-20a-5p predicted in liver hepatocellular carcinoma (LIHC-US) (rows: dysregulated targets; columns: samples with cis-regulatory mutations associated with hsa-mir-20a-5p). The top color scale represents the gene regulatory status posterior probability (red: up-regulation; blue: down-regulation - posterior probability * (-1)). The horizontal bar below shows the miRNA expression z-value (Materials and Methods). (**C**) KEGG 2021 most enriched terms (rows) for all the dysregulated genes associated with the identified miRNA drivers across TCGA cohorts (columns). Terms are ordered by their mean rank across all cohorts. Significance is provided as –log_10_(*P*-value).

Applying this analysis to the seven TCGA cohorts, we predict 68 mature miRNAs, derived from 47 pre-miRNAs, as associated with mutations in TFBSs and deregulation of expression for their target genes (Figure 5A and Supplementary Figure S18; Additional File 3). From these 68 miRNAs, 54 are already annotated as cancer-associated miRNAs in the miRCancer database (71) (*P*-value = 5e–23; hypergeometric test), which is derived from text-mining of the scientific literature in PubMed (76). Moreover, miRCancer provides information about the cancer types that are associated with miRNAs in the literature; ∼27% cancer-associated miRNAs we predict are supported by the literature to be involved in the same cancer type as the cohort from which they were identified (*P*-value = 3.97e–14; hypergeometric test).

Among these, we identify a core set of 12 mature miRNAs (derived from 7 pre-miRNAs) that are identified in at least four out of the seven cohorts (Figure 5A and Supplementary Figure S18): hsa-miR-20a-3p, hsa-miR-92a-1-5p (predicted in all seven cohorts), hsa-miR-18a-5p (six cohorts), hsa-miR-20a-5p, hsa-miR-18a-3p, hsa-miR-17-5p, hsa-miR-17-3p, hsa-miR-155-5p (five cohorts), hsa-miR-155-3p, hsa-miR-708-3p, hsa-miR-708-5p and hsa-miR-205-5p (four cohorts). All these miRNAs are derived from precursors of already established oncomiRs or tumor suppressor miRNAs, or are known to be involved in immune response or inflammation (77–88). Note that hsa-miR-17-3p, hsa-miR-17-5p, hsa-miR-18a-5p, hsa-miR-18a-3p, hsa-miR-20a-3p, hsa-miR-20a-5p and hsa-miR-92a-1-5p are part of a single miRNA cluster on chromosome 13 and this polycistronic cluster (known as miR-17-92) is well known to be composed of oncomiRs involved in proliferation and tumor angiogenesis as well as reducing apoptosis of cancer cells (77).

When visualizing the dysregulated networks of miRNA targets in samples harboring cis-regulatory alterations associated with the predicted cancer-associated miRNAs, we detect subsets of the networks as up- or down-regulated across patients from the same cohort (Figure 5B). The functional pathways are similar to those detected with protein-coding gene networks (Figure 4B-C and 5C). Note that the miRNA-target networks observed with altered expression for a given miRNA may vary between cohorts for the same miRNA because some targets are specifically expressed or altered in a subset of tissues or cell types (Supplementary Figure S19).

Similar to our previous observations with protein-coding genes, miRNA targets with altered expression downstream of cis-regulatory mutations are enriched for transcriptional activity terms and in biological pathways associated with carcinogenesis (Figure 5C). Furthermore, these networks are recurrently found when considering disrupted target genes in each cohort independently (Supplementary Figures S14–S17). We discover several virus infection-related terms enriched across the cohorts (Figures 4B-C and 5C), arguing for a potential link between viral infections and cancer initiation/progression, as previously suggested (89,90), via miRNAs.

Altogether, this study provides the first foray into the analysis of a combined effect of coherent transcriptional and post-transcriptional dysregulation downstream of somatic cis-regulatory mutations associated with miRNAs in cancer cells. It highlights a core set of miRNAs associated with cis-regulatory mutations that are linked to a cascading alteration of gene regulatory networks involved in cancer onset and progression.

### Complementary analysis of an independent breast cancer cohort supports dysregulation of specific pathways

Further, we aim to validate the recurrence of the predictions for breast cancer obtained from the 92 samples of the BRCA-US cohort from TCGA in a complementary cohort. We apply the same methodology with the same parameters to the ICGC breast cancer cohort (43), which is composed of 256 breast cancer samples with the same trio of data types available (WGS, RNA-seq, and miRNA expression - from microarrays; Additional file 4).

Similar to the BRCA-US analysis on protein-coding genes, our analysis of the ICGC cohort predicts known cancer drivers identified by associating LoF or cis-regulatory mutations with dysregulation of their respective gene networks. Breast cancers can be categorized into estrogen receptor positive (ER+) and negative (ER–), each subtype harboring a distinctive signature of gene expression with prognostic and predictive impact. We explore how the distribution of ER status in patients from the two cohorts can impact the predictions of cancer-associated genes. The BRCA-US cohort is composed of approximately the same number of ER+ and ER– patients while the ICGC cohort is composed of 72% of ER+ patients. Given the size of the ICGC cohort (256 samples), it is possible to perform two additional analyses on ER+ (184 samples) and ER– samples (72 samples) independently. The analysis of cis-regulatory mutations associated with protein-coding genes reveals two predictions specifically common to BRCA-US, ICGC, and ICGC ER+ cohorts (*IL12RB1* and *TOP1*), one specifically common to BRCA-US and ICGC (*B4GALT3*), one specifically common to BRCA-US and ICGC ER+ (*CTSS*), and three common to ICGC and ICGC ER– (*MEF2A*, *RB1* and *RGS1*) (Supplementary Figure S20). Out of these seven genes, four are known cancer-associated genes (*B4GALT3*, *CTSS*, *RB1* and *TOP1*). Despite this small intersection, the functional enrichment analyses of the dysregulated genes associated with all predicted genes are similar in the cohorts (Supplementary Figure S21), suggesting that although the predictions vary among cohorts with different etiology, the dysregulated pathways are likely the same. Furthermore, we detect enrichment of similar key cancer pathways when considering the dysregulated genes associated with the predicted cancer-associated genes (Supplementary Figure S21).

**Figure 6.**
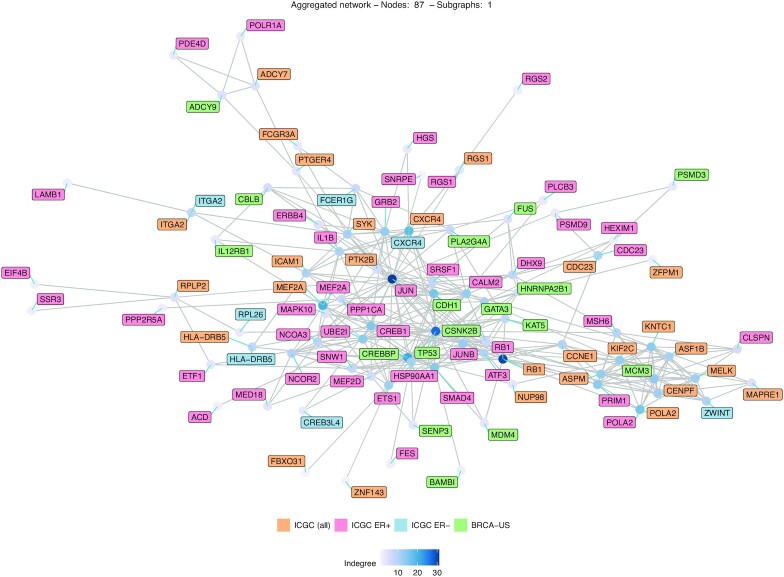
Predicted genes in breast cancer cohorts are connected in the biological network. Network representing the predicted protein-coding genes in ICGC (all samples), ICGC ER+, ER– and BRCA-US cohorts. The names of the genes predicted in two or more cohorts are displayed several times with different colors.

To confirm whether common pathways are deregulated despite the prediction of different genes, we construct the network of all the predicted genes when considering patients from BRCA-US, ICGC, ICGC ER+ and ICGC ER–. Genes are linked in the network if they are known biological partners in the original network (Figure 6). The constructed network comprises 87 genes, which are all connected in a single dense network, where the top three (hub) genes with the largest in-degree are *JUN*, *RB1* and *TP53*. This observation highlights that the predicted genes across the cohorts are likely involved in similar biological pathways, which is supported by the functional enrichment results above. It suggests that the same pathways tend to be dysregulated through mutations associated with different genes.

We predict one miRNA (hsa-mir-378a-3p) associated with cis-regulatory mutations in the ICGC cohort when considering all samples (Supplementary Figure S22). We do not predict any driver miRNAs associated with cis-regulatory mutations when examining specifically the ER+ samples. However, we identify hsa-mir-17-3p, hsa-mir-17–5p, hsa-mir-18a-5p, hsa-mir-20a-5p, hsa-mir-21–5p, hsa-mir-155–5p, hsa-mir-590-5p, and hsa-mir-629-3p when considering ER- samples. Out of these eight miRNAs, two are predicted in the BRCA-US cohort (hsa-mir-17-3p and hsa-mir-18a-5p; Supplementary Figure S22) and five are recurrently found in at least 5 out of the 7 TCGA cohorts (Figure 5 and Supplementary Figure S22). As expected, these results confirm that the cohort clinicopathological composition impacts the predictions as it can impact the landscape gene expression distributions across samples. Nevertheless, the complementary analyses of the BRCA-US and ICGC breast cancer cohorts exhibit hsa-mir-17-3p and hsa-mir-18-5p as recurrently predicted breast cancer-associated miRNAs linked to cis-regulatory mutations and dysregulation of their target gene networks. Functional enrichment analysis confirms that the dysregulated miRNA target gene networks are enriched for genes involved in transcriptional regulation and cancer-relevant pathways such as the p38 MAPK signaling, ErbB signaling, and DNA damage response (Supplementary Figure S23).

**Figure 7.**
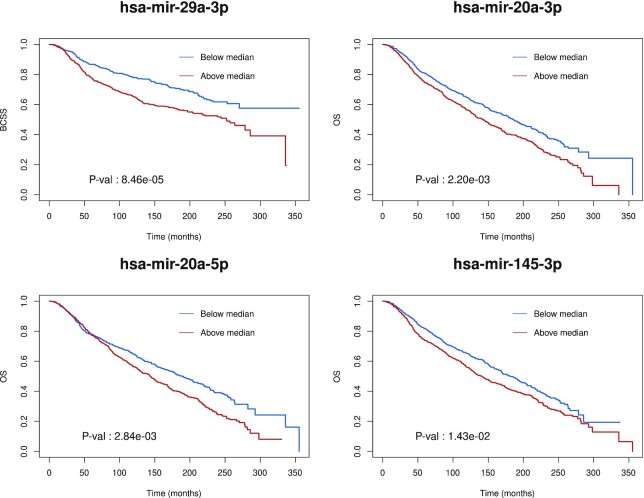
Survival curve analysis for some predicted miRNA drivers. Kaplan–Meier survival curves were obtained using the METABRIC cohort for the most significant driver miRNAs identified in the breast cancer cohorts. Samples were separated into two groups according to the level of miRNA expression (above/below the median). Log-rank test p-values are indicated. OS: overall survival. BCSS: breast cancer-specific survival.

Finally, we evaluate the clinical potential of the predicted breast cancer miRNAs for breast cancer survival estimation. For this purpose, we consider a third cohort, METABRIC (72), which is composed of 1282 samples. We compute Kaplan–Meier survival curves and log-rank tests using miRNA expression from the METABRIC cohort for the miRNAs predicted as drivers in the BRCA-US and ICGC cohorts (for 26 of the predicted miRNAs in breast cancer). Examining both overall survival and breast cancer-specific survival values, we observe log-rank test *P*-values <0.05 for hsa-mir-29a-3p, hsa-mir-20a-5p, and hsa-mir-20a-3p (Figure 7 and Supplementary Figures S24 and S25). Note that hsa-mir-20a-5p and hsa-mir-20a-3p are recurrently predicted in at least five out of the seven ICGC cohorts. Taken together, these results reinforce *a posteriori* the biomarker potential of some miRNAs we predicted as their level of expression could be used for prognosis.

## DISCUSSION

In this study, we explore how cis-regulatory somatic mutations at TFBSs can be used to predict genes with a cascading trans-effect on gene regulatory network dysregulation. Contrary to most methods that predict cancer-driving events based on the recurrence of mutations, we seek to couple cis-regulatory mutation information with gene expression data from the same samples to highlight direct evidence of the regulatory impact of the mutations. By integrating whole-genome somatic mutations, RNA-seq, small RNA-seq, and copy number aberrations (CNA) data with gene regulatory networks, we perform pan-cancer predictions of protein-coding and miRNA genes associated with somatic cis-regulatory mutations in patients from seven distinct cancer types. Our study provides a large-scale foray into predicting cancer-associated protein-coding and miRNA genes by combining both transcriptional and post-transcriptional information. Our results provide new insights into the potential impacts and causes of the alterations of gene regulatory programs observed in cancer cells along with the cascading effects on key biological pathways.

We specifically focus on somatic mutations that reside within a high-quality dataset of TFBSs that represent direct TF-DNA interactions, which cover ∼2% of the human genome, with both experimental and computational evidence (38). We acknowledge that this set of TFBSs might represent a limited subset of all potential TFBSs in the human genome as it was derived from experiments available for a reduced number of TFs and cell types/tissues (231 TFs out of the ∼1600 human TFs reported (11) and 315 cell types and tissues). Moreover, some TFBSs might not be relevant or functional in the cell type of origin associated with the cancer types studied here. Nevertheless, we provide evidence that the regions considered are likely enriched for functional genomic elements since they harbor mutation rates similar to what is observed in exonic regions (Figure 1). This observation is complementary to other studies that showed similar mutation rates in promoters and enhancers compared to protein-coding exons (12,91) and a negative selection for cancer mutations at TFBSs (92). The reduced mutation rates in exons and the limited increase in surrounding regions can be attributed to increased mismatch repair and nucleotide excision repair in exons as previously shown (93,94). The decreased mutation rates when considering TFBSs are in line with our previous observation in B-cell lymphomas (27). Nevertheless, it is somewhat in disagreement with previous studies showing that nucleotide excision repair is impaired by the binding of TFs to DNA (95,96). We hypothesize that the differences observed could be partially explained by the fact that (i) our mutation rate analysis considered TFBSs predicted from several cell lines and tissues instead of focusing on TFs and TFBSs specific to the considered cell types or conditions (such as UV-exposure in melanoma) and (ii) we do not filter TFBSs based on open chromatin data from matched cell types.

Contrary to previous studies assessing the impact of mutations on TF-DNA binding affinity or the enrichment for mutations in cis-regulatory regions (97–100), we particularly evaluate the impact of cis-regulatory mutations on expression alteration in gene networks. As such, our approach does not quantify the direct impact of individual mutations on the obliteration of TF–DNA interactions but uses RNA information as the ultimate readout. Although other features can be used to highlight variants of interest, it has previously been shown that machine learning methods used to assess the effect of mutations on TF binding affinity poorly predict the effects on expression as reported by massively parallel assays (101). A previous method systematically assessed the potential impact of somatic mutations in genomic tiles near genes’ TSSs on gene expression (25). Here, we consider mutations lying within a specific set of pre-defined TFBSs without restrictions on distances to TSSs and evaluate the trans-association of the mutations with genes’ network deregulation. Our approach is somewhat similar to a genome-wide association study framework focused on TFBSs to reduce the search space. Moreover, our strategy is not directly assessing the effect on TF-DNA interactions, i.e. the gain/loss of TFBSs, but rather focuses on the association with gene expression deregulation. Although we focused on somatic mutations and small indels at cis-regulatory elements, we acknowledge that CNAs such as duplications or deletions are likely to contribute to gene expression alteration as well. Nevertheless, our analyses considered CNAs to ensure that the predicted deregulations were not confounded with CNAs. Further work and a complementary computational framework will be necessary to bring together single nucleotide variants, small indels, CNAs, and structural variations and assess their combined impact on gene expression deregulation in cancer.

The analysis of protein-coding genes predicts 28 genes in at least two (out of the seven) TCGA cohorts analyzed, with many already known cancer drivers (Figure 2A). We observe that the protein-coding genes predicted through the analysis of cis-regulatory mutations generally do not contain mutations in exonic regions for the same patients (Figure 2B and Supplementary Figure S12). This observation suggests complementary mechanisms acting upon gene expression dysregulation with cascading effects on regulatory network disruption. We hypothesize that either the final product of a gene may be altered due to LoF mutations or the expression of the gene is altered through cis-regulatory mutations, which, in both cases, alter the activity of biological networks.

Given that miRNAs cover a small portion of the human genome, they harbor a small number of somatic mutations (8), limiting the possibility to affect gene expression. The potential mechanism that we propose here is the alteration of their regulatory elements. Our study highlights cis-regulatory mutations linked to miRNAs that are associated with dysregulation of expression of the miRNA targets. In our pan-cancer analysis, we discover a core set of 12 mature miRNAs associated with the dysregulation of key pathways involved in carcinogenesis. This core set of miRNAs represents a common feature for gene expression dysregulation associated with cancer onset or progression. We note that several of these miRNAs are established oncomiRs, which promote carcinogenesis. The Kaplan–Meier plots in Figure 7 for hsa-mir-29a-3p, hsa-mir20a-3p, hsa-mir-20a-5p, and hsa-mir-145-3p show that higher expression correlates with poorer survival rates, which would indicate that these miRNAs act as oncomiRs in breast cancer, possibly targeting tumor suppressor genes or pathways.

The analysis of the dysregulated networks of the predicted cancer-associated genes (protein-coding and miRNAs) shows that many genes are dysregulated in a few samples but rarely across all the mutated samples (Figure 5B). However, the functional enrichment analysis of the dysregulated genes shows consistency across cohorts and the analyzed types of mutations (LoF and cis-regulatory) for both protein-coding and miRNA genes, even when there is a small intersection among the predicted genes in cohorts of the same cancer type (Supplementary Figures S20 and S21). Altogether, these observations suggest a phenotypic heterogeneity (i.e. alterations of different parts of the same network lead to the same phenotype), which may have originated because the dysregulated genes are connected in the biological network (Figure 6). Moreover, as originally described in Ding *et al.* (26), the *xseq* probabilistic framework highlights the specific samples where mutations are associated with an impact on gene expression (Figure 4A). This dichotomy can, in principle, be used to stratify samples and mutations but, in this study, is limited by the number of samples considered.

We apply our methodology to two cohorts of breast cancer samples (BRCA-US and ICGC). Given the large number of samples in ICGC (*n* = 256), we perform three analyses separately by considering (i) all samples, (ii) ER+ samples and (iii) ER– samples. Predictions vary depending on the samples’ histopathology. This is particularly important for methods relying on gene expression, which is influenced by the clinical composition of the cohorts. We acknowledge that methodological differences between the BRCA-US and ICGC cohorts (e.g. different somatic mutation calling algorithms, RNA-seq versus microarrays, and normalization of RNA-seq raw counts) can provide additional explanations for the variation in predictions, which is the case with the BRCA-US and ICGC cohorts that were independently normalized. Although only a few of the predicted protein-coding genes are predicted in both the ICGC and the BRCA-US cohorts (Supplementary Figure S20), the functional enrichment analysis of the dysregulated gene networks is consistent (Supplementary Figure S21). This observation suggests common dysregulated pathways that act as attractors and that could originate from (non-recurrent) distinct cancer-associated events. It underlines the importance of addressing cancer as a disease with perturbations manifested at the gene network level. Our miRNA analyses target gene expression alteration recurrently altered across the BRCA-US and ICGC ER– breast cancer cohorts and highlight two miRNAs (hsa-mir-17-3p and hsa-mir-18-5p) associated with cis-regulatory mutations.

Despite the multiple lines of evidence for the prediction of cancer-associated genes in this study, we acknowledge that the predictions can provide false positives and false negatives due to multiple reasons such as: (i) a limited number of TFs with high-quality TFBSs; (ii) TFBS-target gene associations obtained by a naive approach combining information from an integrative database (56) and association to the closest TSS (Supplementary Figure S8)—we hypothesize that many of these associations may be irrelevant or incorrect and many others are missing; (iii) a diversity of tumor purity within the considered samples, despite the original threshold of 80% used by TCGA; (iv) a limited number of WGS datasets (tens of samples) within each cohort, compared to the number of samples with WXS (hundreds) used in other studies; (v) prior networks that might be incomplete or with incorrect associations. Importantly, one of the main limitations of this project is the low number of tumor samples with both WGS and RNA-seq data; this limitation not only biases the community research toward the study of exonic regions but also limits the statistical power of the methods assessing the impact of cis-regulatory mutations on gene network expression alteration.

Altogether, we argue that our capacity to predict cancer-associated cis-regulation mutations will increase as more high-quality TFBSs for more TFs and improved methods to associate TFBSs with their target genes become available. In addition, focusing on cis-regulatory regions specifically open or active in cancer samples would inform where somatic mutations are likely effective. We expect that with more WGS, RNA-seq and other genomics datasets derived from cancer samples available, the community will revert the paucity in the detection of noncoding cancer-associated events (8).

## CONCLUSION

By integrating whole-genome somatic mutations, RNA-seq, and small RNA-seq data with gene regulatory networks across seven cancer types, we identify cis-regulatory mutations associated with the dysregulation of gene regulatory networks through specific protein-coding and miRNA genes. The enrichment for known cancer-associated genes and the functional enrichment analysis reinforce a posteriori the predicted protein-coding and miRNA genes as being involved in biological pathway alteration affecting cancer development through exonic and cis-regulatory alterations. Our study represents, to our knowledge, the first large-scale analysis of cis-regulatory mutations that are linked to gene expression alteration in key cancer-associated pathways. Our results suggest that this process can be achieved flexibly because although we observe different genes in different patients, all are associated with deregulation of the same pathways. Combining transcriptional and post-transcriptional information, we identify a core set of 12 miRNAs linked to altered cancer pathways across cancer types. These pan-cancer results provide new insights into the impact and potential causes of miRNA-mediated gene expression dysregulation. This work extends our capacity to address the discovery gap of cancer-associated event identification through the analysis of noncoding mutations and miRNA genes.

## DATA AVAILABILITY

The analysis with all the scripts and parameters can be found through the following link: https://bitbucket.org/CBGR/workspace/projects/DYS. We provide (i) the source code for the analysis and (ii) a pipeline for users to run similar analysis with their own data. The repositories can be accessed with the following links: for the dysmiR pipeline: https://bitbucket.org/CBGR/dysmir_pipeline, for the manuscript: https://bitbucket.org/CBGR/dysmir_manuscript.

## Supplementary Material

gkac1143_Supplemental_Files
