# Supplementary material for: Cis-regulatory mutations associate with transcriptional and post-transcriptional deregulation of gene regulatory programs in cancers
Source: Nucleic Acids Res. 2022 Dec 8;50(21):12131–48. doi: 10.1093/nar/gkac1143 (PMC9757053; doi:10.1093/nar/gkac1143)
Supplement: gkac1143_Supplemental_Files [file gkac1143_supplemental_files.zip › Additional_files_captions.pdf]

Additional file 1. **TCGA-selected samples (specimens) with their ICGC/TCGA IDs.** Samples were selected to provide WGS, RNA-seq, small RNA-seq, and CNA data. All samples considered in this study correspond to solid tumors.

Additional file 2. **Summary of the number of mutations in the TCGA cohorts.** Basic summary statistics (median, mean, and standard deviation) of the total number of mutations and the number of mutations overlapping TFBSs.

Additional file 3. **Predicted genes (miRNAs and protein-coding genes) with their dysregulated targets.**

Additional file 4. **ICGC selected samples and their ER status.** Samples were selected to provide WGS, RNA-seq, miRNA microarray expression, and CNA data.

Additional file 5. **Description of the resources used for this manuscript.** Brief description of the resources (gene lists, databases, external files) used to perform this study, with links to their corresponding sites and the date when they were downloaded.
